# Expression of 5α- and 5β-reductase in spinal cord and muscle of birds with different courtship repertoires

**DOI:** 10.1186/s12983-016-0156-y

**Published:** 2016-06-10

**Authors:** Matthew J. Fuxjager, Eric R. Schuppe, John Hoang, Jennifer Chew, Mital Shah, Barney A. Schlinger

**Affiliations:** Department of Biology, Wake Forest University, 228 Winston Hall, Winston-Salem, NC 27109 USA; Center for Molecular Communication and Signaling, Wake Forest University, Winston-Salem, USA; Department of Integrative Biology and Physiology, University of California, Los Angeles, USA; Laboratory of Neuroendocrinology, Brain Research Institute, University of California, Los Angeles, USA; Department of Ecology and Evolutionary Biology, University of California, Los Angeles, USA; Smithsonian Tropical Research Institute, Balboa, Ancon, Republic of Panama

**Keywords:** Hormones, Androgens, Testosterone androgen receptor, Courtship behavior, Elaborate displays, Reproduction, Tropical birds, Manakins, Neuromuscular system, Skeletal muscles

## Abstract

**Background:**

Through the actions of one or more isoforms of the enzyme 5α-reductase in many male reproductive tissues, circulating testosterone (T) undergoes metabolic conversion into 5α-dihydrotestosterone (DHT), which binds to and activates androgen receptors (AR) with greater potency than T. In birds, T is also subject to local inactivation into 5β-DHT by the enzyme 5β-reductase. Male golden-collared manakins perform an androgen-dependent and physically elaborate courtship display, and these birds express androgen receptors in skeletal muscles and spinal cord at levels far greater than those expressed in species with more limited courtship routines, including male zebra finches. To determine if local T metabolism facilitates or impedes activation of male manakin courtship, we examined expression of two isoforms of 5α-reductase, as well as 5β-reductase, in forelimb muscles and spinal cords of males and females of the two aforementioned species.

**Results:**

We found that all enzymes were expressed in all tissues, with patterns that partially predict a functional role for 5α-reductase in these birds, especially in both muscle and spinal cord of male manakins. Moreover, we found that 5β-reductase was markedly different between species, with far lower levels in golden-collared manakins, compared to zebra finches. Thus, modification to neuromuscular deactivation of T may also play a functional role in adaptive behavioral modulation.

**Conclusions:**

Given that such a role for 5α-reductase in androgen-sensitive mammalian skeletal muscle is in dispute, our data suggest that, in birds, local metabolism may play a key role in providing active androgenic substrates to peripheral neuromuscular systems. Similarly, we provide the first evidence that 5β-reductase is expressed broadly through an organism and may be an important factor that regulates androgenic modulation of neuromuscular functioning.

## Background

Sex steroid hormones activate diverse vertebrate reproductive behaviors by their actions on central and peripheral tissues. In reproductively active males, gonadal testosterone (T) increases aggressive behavior, as well as appetitive (courtship) and consummatory (copulatory) behavior. To achieve these effects, T acts on neural circuits and/or skeletal muscles that motivate behavioral performance and enhance motor output [1–3]. The actions of T often require its metabolic conversion into one or more active products. In brain, for example, T is converted into estradiol by the enzyme aromatase; in turn, this estrogen acts via estrogen receptors on discrete neural circuits to facilitate the performance of copulatory and aggressive behaviors, as well as a variety of other neural functions [1].

In many male reproductive tissues, T undergoes conversion into the potent androgen 5α-dihydrotestosterone (5α-DHT) by one of two main isoforms (types 1 and 2) of the enzyme 5α-reductase [4–6]. This 5α-DHT binds strongly to androgen receptors (AR) and thereby impacts tissues involved with reproductive function and the masculine phenotype. When T acts via AR to mediate the performance of male copulatory behavior, for example, there is good evidence that local 5α-reductase within central neural circuits plays a crucial role [7–11]. Although AR activation in brain has been shown to underlie the performance of some male appetitive behaviors, such as aspects of courtship [12–16], much less is known about the role of 5α-reductase in androgen-dependent peripheral neuromuscular systems [17, 18]. In skeletal muscle in particular, AR is often expressed and functional [19–28], but there remains some uncertainty, especially in humans, as to whether 5α-reductase is expressed sufficiently to influence the actions of circulating T [29–32].

In other tissues of some species, especially birds, T can undergo conversion into an inactive androgen, 5β-dihydrotestosterone (5β-DHT), by the enzyme 5β-reductase [6]. Although studies of this enzyme are limited compared to those investigating 5α-reductase, research over the past decades suggests that 5β-reduction of androgens can locally regulate steroid hormone action. For example, 5β-reductase acts in the avian hypothalamus to regulate the production of androgen-dependent sexual behavior [33]. This enzyme is present in a variety of brain regions in developing birds, where it is thought to help guide sexual differentiation and masculinization of sexually dimorphic neural systems by locally regulating the availability of T or its bioactive metabolites [34–37]. Together, this work leads to the hypothesis that 5β-reductase outside of the brain may similarly adjust androgenic metabolism to guide how and where androgens act on extra-neuronal target tissues that influence sexual interactions between conspecifics.

To explore a potential role for actions of 5α-reductase on peripheral neuromuscular systems, we have investigated the expression of types 1 and 2 of 5α-reductase in the spinal cord and in skeletal muscles of two avian species, the golden-collared manakin (*Manacus vitellinus*) and the zebra finch (*Taenoygia guttata*). Male manakins perform a complex, physically intensive courtship routine that involves elaborate dances above the forest floor and the production of mechanical wing-snaps [38, 39]. Recent research identifies a variety of physiological adaptations that underlie this ability to produce athletic sexual signals [40–43], with a variety of studies showing that the production of such behavior depends upon androgen action via AR, especially in the periphery [41, 44–46]. In this species, AR are expressed in significant amounts in both motor and sensory spinal neurons, as well as in skeletal muscles involved in lifting (*supracoracoideus*, SC), rotating/retracting (*scapulohumeralis caudalis*, SH), and depressing (*pectoralis*, PEC) the wings, which are prominent movements of their physical courtship displays [41, 47]. Across spinal cord regions and muscles, AR are expressed at similar levels in male and female manakins [41, 47], but males accumulate more radioactivity in the spinal cord than do females after injection of radioactive T [48]. Recently, we found that T-dependent changes in gene expression profiles vary across the wing muscles in males [28], despite equivalent expression of AR [41, 44, 47]. Both of these effects could result from differences across sex or muscle in T metabolism, such as in the abundance of 5α and/or 5β-reductases.

Likewise, male zebra finches perform a physically modest courtship display [49]. Compared to the manakin, zebra finches express AR significantly at lower levels of AR in both their spinal cord and wing muscles [41, 44, 47]. Further activation of AR in zebra finch and manakin muscles induces different transcriptional changes that presumably produce distinct functional effects on whole muscle performance [28]. These differences in the effect of androgens may stem from species variation in expression of 5α and/or 5β-reductases.

To provide 5α-DHT to facilitate the performance of physical courtship in manakins, we hypothesize that type 1 and/or type 2 5α-reductase will be expressed at higher levels in spinal cord and in skeletal muscle of males, as compared to female manakins and zebra finches of both sexes. As birds are unique in expressing significant amounts of the enzyme 5β-reductase in many of their tissues [33, 34, 37, 50], we also hypothesized that female manakins and zebra finches will express this enzyme in greater amounts than males to reduce T activation.

## Methods

### Animals

The appropriate governmental agencies and the Animal Care and Use Committees of the University of California, Los Angeles (UCLA) and the Smithsonian Tropical Research Institute (STRI) approved the procedures described herein below. Using passive mist netting, adult male (*n* = 4-7) and female (*n* = 5) golden-collared manakins were captured from courtship leks in forests near the village of Gamboa in central Panama. Birds were caught at the height of the breeding season during March and April. Males were captured while courting females in the leks, while females were captured while visiting individual males; thus, all manakins used in this study were reproductively active.

Adult male (*n* = 6) and female (*n* = 5) zebra finches were obtained from our laboratory colony at UCLA (light cycle: 14 h light, 10 h dark), where they were housed in adjacent same-sex, open-flight aviaries (6′ × 6′ × 3′). The birds were in visual and acoustic contact so that courtship occurred between individuals of the opposite sex. Indeed, many of the males were observed actively singing toward the female cage, and thus were in the necessary reproductive condition to solicit copulations.

All birds included in these studies were euthanized via rapid decapitation, at which point the spinal cord was dissected and separated into cervical, lumbar, and thoracic components. The cervical component was characterized as bottom of cervical enlargement to the brainstem, whereas the lumbar component was characterized as the top of the lumbosacral enlargement to the terminus of the cord. The thoracic cord was characterized as the spinal cord in-between the cervical and lumbar sections. At this time, we also dissected the SC, SH and PEC muscles. Once the tissue was removed, it was immediately flash frozen on dry ice, and stored at −80 °C as described previously [27, 28, 41, 44].

During tissue dissection, we verified that the gonads of male birds were enlarged in a manner consistent with that of an actively breeding individual. This information, along with observations of male courtship prior to euthanization (see above), indicates that circulating androgen levels were elevated [51–53]. Furthermore, past work shows that, under these seasonal or photoperiod conditions, testosterone levels are basal in females of both species [51, 53].

### PCR

Muscle and spinal cord samples were homogenized for 30 s at medium speed using a rotor/sator homogenizer. Total RNA was isolated with Trizol® (Invitrogen, Carlsbad, CA) according to the manufacturer’s instructions, and RNA concentrations were determined using a Nanodrop system (Thermo Scientific, Wilmington, DE, USA).

For each sample, 1 μg of RNA was first treated with DNAse and then reverse transcribed using Superscript II Reverse Transcriptase (Invitrogen). The latter reaction occurred at 42 °C for 50 min and then at 70 °C for 15 min. The resultant cDNA was used for PCR amplification of the SRD5A1, SRD5A2, and SRD5B1 genes to confirm that they were indeed expressed in the harvested manakin and zebra finch tissues. Primers for these genes were designed using the annotated zebra finch genome (Table 1), and PCR reactions contained 50 ng of cDNA, 0.06 ng of DNA taq polymerase, 0.4 μM of forward and 0.4 μM of reverse primer, dNTP, and buffer. Reactions were first run at 95 °C for 5 min and then followed by 38 cycles of 95 °C for 30 s, 60–65 °C for 30 s, and 72 °C for 1 min. Reactions were completed at 72 °C for 10 min. PCR products were run on a gel to ensure that the size of the amplified fragment matched the expected size, and a subset of products were sequences (Genewiz Inc., La Jolla, CA, USA) for further validation.

**Table 1:** Primer sequences for PCR designed from Zebra Finch genome

Gene	Direction	Sequence	Amplicon size (bp)
SRD5A1	Forward	TTTCACTTTTGTGTTAGCACTTCTG	403
	Reverse	TGGATAGTCTTCAAATTTCTCAAGG	
SRD5A2	Forward	CCTTTCTTCACTAGAGGCAGACC	435
	Reverse	TGGATAGTCCGTAAATGTCTTGAG	
SRD5B1	Forward	AGAAAACTCCCAAAGGTTCCTG	401
	Reverse	TGCCAAGCCTGCATCTTTAC	

### Quantitative PCR (qPCR)

Procedures used for qPCR on avian muscles are described in detail elsewhere [27, 41, 44, 47]. Reactions were performed in an ABI 7300 sequence detection system, using SYBR Green Master Mix kits (Applied Biosystems, Inc., Foster City, CA). Each reaction contained 100 ng of template, 0.9 μM of forward and 0.9 μM of reverse primers. For each gene, we developed species-specific primers based on the amplified fragments obtained through PCR sequencing (Table 2). Primers for the internal control gene – glyceraldehyde-3-phosphate dehydrogenase (GAPDH) – were based on the annotated zebra finch genome and were used for both species (Forward, 5′-TGACCTGCCGTCTGGAAAA; Reverse, 5′-CCATCAGCAGCAGCCTTCA, 70-bp product). Our laboratory has verified that GAPDH does not differ in primer binding and reaction efficiency between manakins and zebra finches and thus serves as a useful internal control [27, 44, 47].

**Table 2:** Species-specific quantitative PCR (qPCR) primers

Species	Gene	Direction	Sequence	Amplicon size (bp)
Golden-collared Manakin	SRD5A1	Forward	CCAAGAGGAGGAATGTTTGAG	142
		Reverse	CCCTTGAACCCAAGATGAAA	
	SRD5A2	Forward	AAGATTCCTCAAGGGGGACTG	132
		Reverse	GCAAAGAGTGAAAAAGGCAAAG	
	SRD5B1	Forward	TTTACTGTGGCAAGCTGTGG	75
		Reverse	TCTTCAGTGTCTTCTCCAGTGTG	
Zebra Finch	SRD5A1	Forward	AGGCCGAGCTTACCACCTAT	68
		Reverse	ACCTGTAATGAAGCGCAAGC	
	SRD5A2	Forward	GGCCTCCTCTTATTTCTGCTG	108
		Reverse	TCCTCCTTGGGGAATCTTG	
	SRD5B1	Forward	CTTTCAAGCCTGGAGATGCAC	83
		Reverse	CCAAGTGGCACATAAGTCTGTC	

qPCR reactions were run at 50 °C for 2 min and then 95 °C for 10 min. Each reaction was then subjected to 40 cycles of 95 °C for 15 s and 60 °C for 1 min. This was followed by a dissociation stage, whereby reactions were run at 95 °C for 15 s, 60 °C for 30 s, and 95 °C for 15 s. Reaction efficiencies fell between 90 and 110 %, and dissociation curves were used to verify the absence of contamination. Samples were run in duplicate. The standard curve method was used to measure the relative expression of each gene of interest (i.e., quantity gene of interest/quantity of GAPDH) [54].

### Statistical analysis

To assess differences in mRNA expression of type 1 5α-reductase, type 2 5α-reductase, and 5β-reductase in both golden-collared manakins and zebra finches, we utilized two-way analysis of variance (ANOVA) with sex and muscle or spinal cord region as factors. Significant main effects were further examined with LSD post-hoc analyses, and significant interactions were examined by simple main effect post-hoc analyses. All data were log transformed [log (1 + X)] to meet conditions of normality. Statistical analyses were performed using SPSS v20.

## Results

### PCR

In both manakin and zebra finch, we successfully amplified fragments of the SRD5A1, SRD5A2, and SRD5B1 genes, which encode the type 1 5α-reductase, type 2 5α-reductase, and 5β - reductase, respectfully. Alignment analyses revealed that the gene sequences were highly similar between species, with ≈ 90 % homology for SRD5A1, ≈96 % homology for SRD5A2, and ≈ 96 % homology for SRD5B1. These data suggest that the genes that express androgenic reductase enzymes are relatively conserved between the golden-collared manakin and zebra finch. Furthermore, these data may even imply broader conservation in reductase enzymes among passerines, given that manakins and finches are relatively distantly related within their common Order.

### Golden-collared manakin spinal cord

**Fig. 1:**
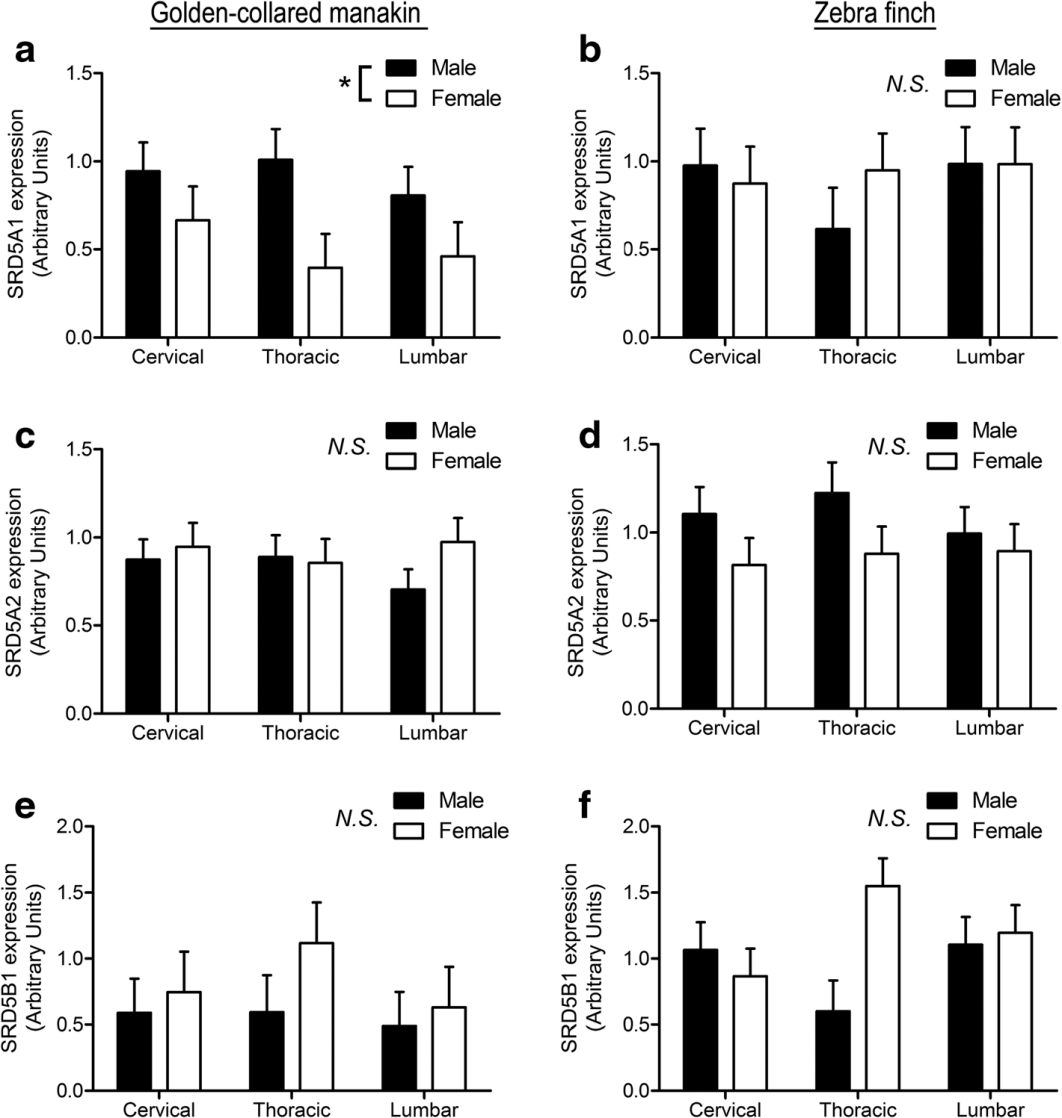
Reductase expression in the spinal cord of golden-collared manakins (left column; *n* = 7 male, *n* = 5 female) and zebra finches (right column; *n* = 6 male, *n* = 5 female). Regions of the spinal cord are denoted on the bottom axis. **a**, **b** Relative expression of the gene for type 1 5α-reductase. **c**, **d** Relative expression of the gene for type 2 5α-reductase. **e**, **f** Relative expression of the gene for 5β-reductase. Data are presented as mean ± 1SEM. Asterisks (*) denote significant differences (*p* < 0.05) in relative expression between sexes (depicted in legend). N.S indicates non-significant difference (*p* > 0.05)

We first examined type 1 5α-reductase expression in the spinal cord of the golden-collared manakin (Fig. 1). We found a significant effect of sex for type 1 5α-reductase expression (F (1,29) = 8.68, *p* = 0.03), with males expressing this transcript more abundantly than females (*p* < 0.05). However, we discovered no regional differences in type 1 5α-reductase across the spinal cord (F (1,29) = 0.52, *p* = 0.60), nor did we find a sex × spinal cord region interaction (F (1,29) = 0.58, *p* = 0.57).

We detected no evidence of significant variation in expression patterns of either type 2 5α-reductase (sex: F (1,29) = 1.14, *p* = 0.30; spinal cord region: F (1,29) = 0.12, *p* = 0.89; sex × spinal cord region interaction: F (1,29) = 0.62, *p* = 0.55) or 5β-reductase (F (1,29) = 1.60, *p* = 0.22; spinal cord region: F (1,29) = 0.23, *p* = 0.80; sex × spinal cord region interaction: F (1,29) = 0.04, *p* = 0.96).

### Zebra finch spinal cord

We also examined enzyme expression in the spinal cords of zebra finches (Fig. 1). Overall, we found no evidence of variation by sex, spinal cord region, or an interaction between these two factors on the expression of type 1 5α-reductase (sex: F (1,23) = 0.13, *p* = 0.72; region: F (1,23) = 0.45, *p* = 0.64; interaction: F (1,23) = 0.68, *p* = 0.52), type 1 5α-reductase (sex: F (1,23) = 3.87, *p* = 0.06; region: F (1,23) = 0.34, *p* = 0.72; interaction: F (1,23) = 0.27, *p* = 0.77), or 5β-reductase (sex: F (1,23) = 1.07, *p* = 0.31; region: F (1,23) = 0.29, *p* = 0.76; interaction: F (1,23) = 1.84, *p* = 0.18).

### Golden-collared manakin muscle

**Fig. 2:**
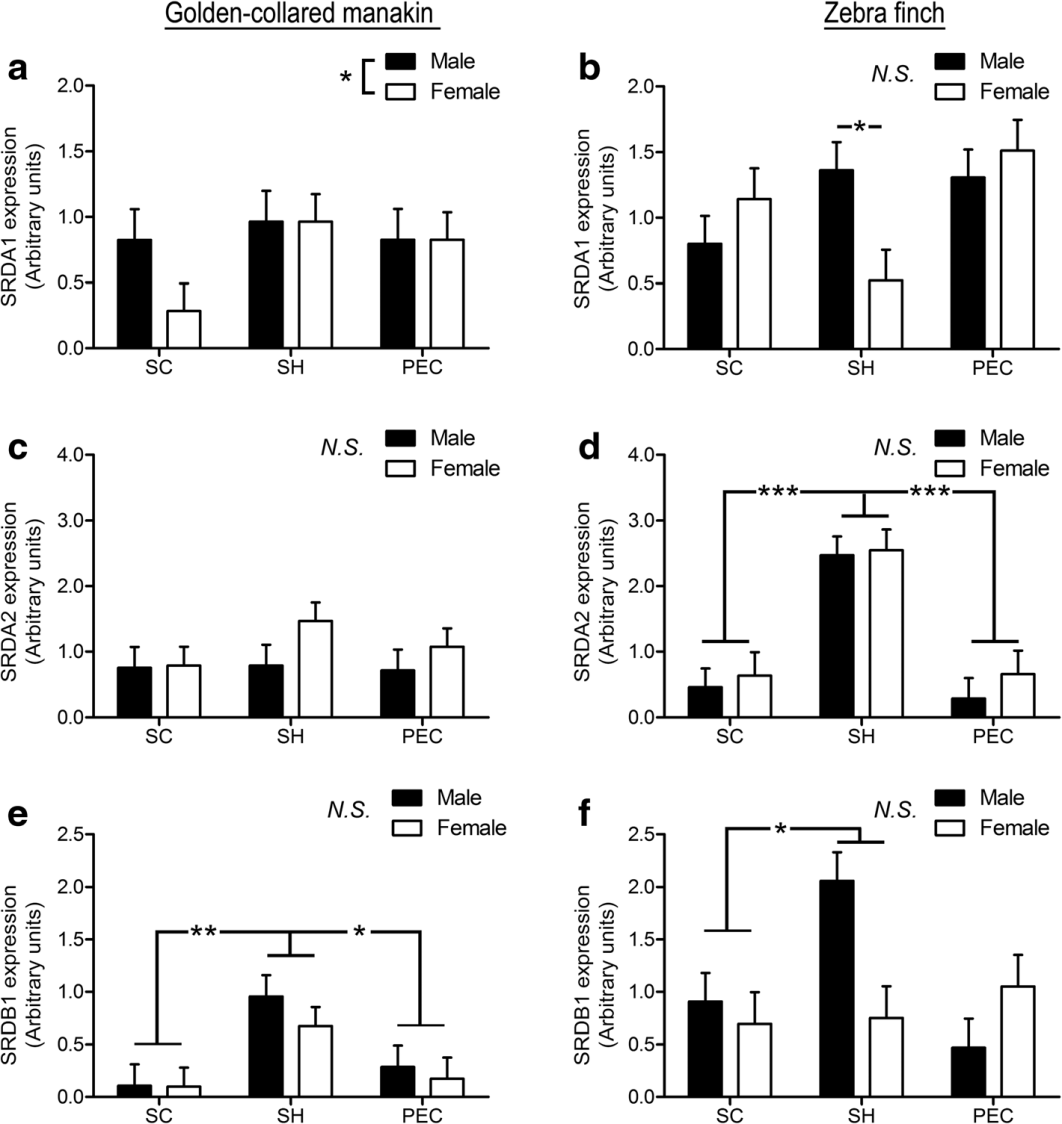
Reductase expression in the wing muscles of golden-collared manakins (left column; *n* = 4 male, *n* = 5 female) and zebra finches (right column; *n* = 6 male, *n* = 5 female). Muscles are denoted on the bottom axis (*supracoracoideus*, SC; *scapulohumeralis caudalis*, SH; and *pectoralis*, PEC). **a**, **b** Relative expression of the gene for type 1 5α-reductase. **c**, **d** Relative expression of the gene for type 2 5α-reductase. **e**, **f** Relative expression of the gene for 5β-reductase. Data are presented as mean ± 1SEM. Asterisks denote significant differences (*=*p* < 0.05; **=*p*<0.01; ***=*p*<0.001) in relative expression between sexes (depicted in legend) or among muscles. N.S indicates non-significant difference (*p* > 0.05)

Next, we examined the expression patterns of reductase enzymes in the main wing muscles of the golden-collared manakins (Fig. 2). We uncovered a significant sex difference in type 1 5α-reductase expression (F (1,20) = 5.65, *p* = 0.03), such that males expressed more of this gene in these muscles than females (*p* < 0.05). There was no difference in type 1 5α-reductase across muscle (F (2,20) = 0.66, *p* = 0.53), nor was there a sex × muscle interaction (F (2,20) = 0.35, *p* = 0.71).

We found that muscle expression of type 2 5α-reductase was statistically indistinguishable across sex (F (1,21) = 2.63, *p* = 0.12) and muscle (F (2,21) = 0.70, *p* = 0.50). Further, there was no evidence of a significant sex × muscle interaction on the expression of this gene (F (1,21) = 0.53, *p* = 0.60).

With respect to the expression of 5β-reductase, levels of this transcript varied among muscles (F(2,20) = 8.40, *p* < 0.01), in that they were significantly higher in the SH compared to either the SC (*p* < 0.001) or PEC (*p* < 0.01). Yet, there was no difference in 5β-reductase mRNA expression between the SC and PEC. We found no sex difference (F(1,20) = 0.80, *p* = 0.38) or sex × muscle interaction (F(2,20) = 0.26, *p* = 0.78) in the expression of this gene.

### Zebra finch muscle

We similarly compared reductase expression patterns in the wing muscles of zebra finches (Fig. 2). For type 1 5α-reductase expression, we found no effect of sex (F (1,27) = 0.32, *p* = 0.58) or muscle (F (2,27) = 3.06, *p* = 0.06) on mRNA levels. However, we did uncover a significant sex × muscle interaction (F (2,27) = 5.33, *p* = 0.01), and further post-hoc tests showed that males maintained higher levels of type 1 5α-reductase in their SH (*p* < 0.05). In the other two wing muscles, expression levels of this gene were indistinguishable between the sexes (*p* > 0.05).

There was an effect of muscle on type 2 5α-reductase expression (F (1,24) = 34.17, *p* < 0.001), such that transcript levels were higher in the SH compared to the SC (*p* <0.001) and PEC (*p* <0.001). Otherwise, we found no difference in on type 2 5α-reductase expression between the sexes (F (1,24) = 0.87, *p* = 0.36), nor did we find evidence of a sex × muscle interaction (F (1,24) = 0.26, *p* = 0.77).

For 5β-reductase, we found that expression levels varied among muscles (F(2,27) = 3.55, *p* = 0.043), with expression being greater in the SH compared to both the SC (*p* < 0.05) and the PEC (*p* < 0.05). Moreover, while there was no overall sex difference in (F(1,27) = 1.32, *p* = 0.26), we did detect a significant sex × muscle interaction (F(2,27) = 6.39, *p* < 0.01). Post-hoc analyses showed that males express more 5β-reductase in the SH than females (*p* < 0.05), but that such differences were not present in either the SC (*p* > 0.05) or the PEC (*p* > 0.05).

### Species comparisons

**Fig. 3:**
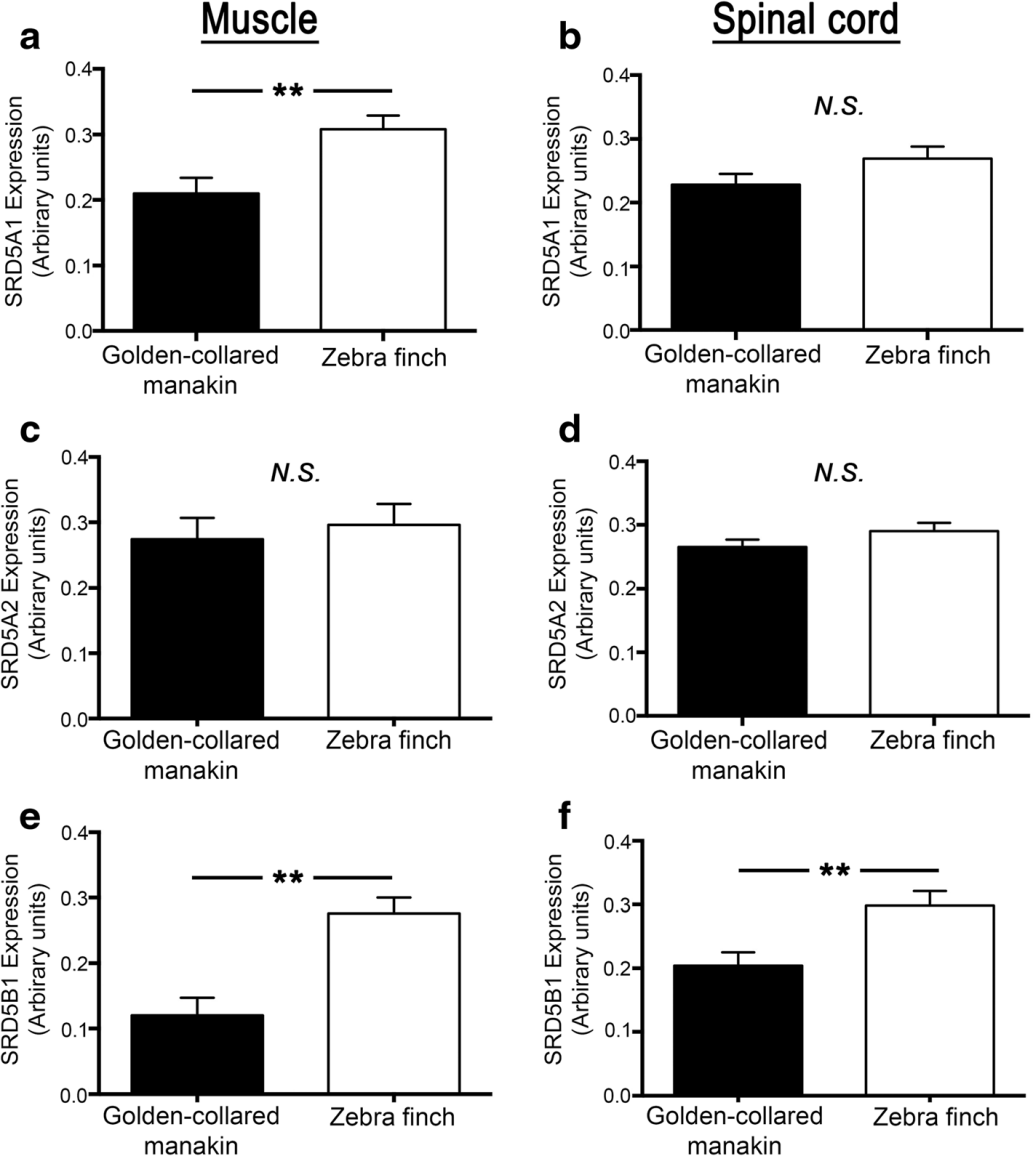
Collective reductase expression in the skeletal muscle (left column) and spinal cord (right column) of golden-collared manakins and zebra finches (denoted on the bottom axes). **a**, **b** Relative expression of the gene for type 1 5α-reductase. **c**, **d** Relative expression of the gene for type 2 5α-reductase. **e**, **f** Relative expression of the gene for 5β-reductase. Data are presented as mean ± 1SEM. Asterisks (**) denote significant differences (*p* < 0.01) in relative expression between species (depicted in legend). N.S indicates non-significant difference (*p* > 0.05)

In a final analysis, we compared expression patterns of reductase enzymes across species (Fig. 3). In the spinal cord, we found no difference between the golden-collared manakin and zebra finch in the expression of either type 1 5α-reductase (F (1,62) = 2.58, *p* = 0.11) or type 2 5α-reductase (F (1,62) = 1.96, *p* = 0.17). However, spinal cord expression of 5β-reductase was significantly greater in zebra finches, compared to golden-collared manakins (F (1,62) = 9.32, *p* = 0.003).

We found that muscle expression of type 1 5α-reductase was greater in zebra finches than in golden-collared manakins (F (1,57) = 9.61, *p* = 0.003). By contrast, expression of type 2 5α-reductase in the muscle was statistically indistinguishable between these two species (F (1,55) = 0.23, *p* = 0.63). Importantly, we also found that levels of muscular 5β-reductase were significantly greater in the zebra finch, compared to the golden-collared manakin (F (1,57) = 18.66, *p* < 0.001).

## Discussion

The general hypothesis motivating this research is that, in both wing muscle and spinal cord, enzymes involved in the synthesis of 5α-reduced androgens are expressed at greater levels in birds that demonstrate significant androgen-dependent motor function as part of masculine courtship displays. Thus, we predicted higher expression of 5α-reductase isoforms not only in male birds compared to females, but also in golden-collared manakins compared to zebra finches. Because 5β-reductase inactivates androgens, we predicted an inverse expression pattern for this enzyme, relative to the activating 5α-reductase isoforms.

Our results provide several lines of evidence, but not all, to support the predictions outlined above. First, we detected expression of both type 1 and type 2 5α-reductase isoforms in all the tissues and in both of the species we examine. Expression of these enzymes suggests that androgen metabolism actively occurs in the spinal cord and wing muscles, effects likely conserved across both oscine and suboscine passerine species. Second, we found that male golden-collared manakins express relatively higher levels of type 1 5α-reductase in their spinal cord and wing muscles, compared to females of this species. This overall sex difference was not present in zebra finches, which instead showed a sex differences in the expression of type 1 5α-reductase only in the SH muscle. Thus, the capacity to form the active androgen 5α-DHT in the manakin spinal tissue and wing musculature may be greater in males than in females, but this sex difference is apparently absent in zebra finches.

Interestingly, zebra finches expressed more type 1 5α-reductase in their muscles than golden-collared manakins, regardless of sex or wing muscle in question. This result is contrary to our prediction, as it suggests that zebra finches have a greater capacity to metabolize T into its more potent state. Androgens do impact zebra finch muscles [27, 28], so increased 5α-reductase may somewhat compensate for the low amount of AR expressed in skeletal muscle [41, 47]. Alternatively, high levels of type 1 5α-reductase in zebra finches may be functionally opposed by the increased expression of 5β-reductase, which may inactivate T locally reducing its availability for conversion to 5α-DHT [33]. Indeed, golden-collared manakins express significantly lower levels of 5β-reductase in both their wing muscles and spinal cord, compared to the zebra finch. This suggests that the manakins’ ability to enzymatically limit androgen action within these neuro-motor systems is reduced, relative to the zebra finch. Such limited inactivation may fundamentally augment the metabolism of T to its 5α-reduced ligand-active state. This interpretation is consistent with previous work that shows that androgens affect the transcription of a greater proportion of the genome in manakins than in zebra finches [28]. Furthermore, these data imply that modulation of androgenic metabolism may occur through alterations of singular – but different – reducing enzymes, as opposed to all reducing enzymes.

Given that the wing muscles constitute a large portion of a bird’s mass, our result showing abundant expression of 5β-reductase in both the manakin and zebra finch indicate that this enzyme is transcribed throughout much of the avian body. Past work in several birds shows that 5β-reductase is located in the brain, pituitary, skin and cloacal gland [33, 50], whereas work in mammals shows 5β-reductase present only in liver [55]. Our study therefore suggests that the enzymatic machinery to reduce androgens to the inactive metabolite 5β-DHT is widespread in bird tissues where it likely fine-tunes the ability of androgens to act throughout the body.

### A role for androgenic metabolism to support physically elaborate displays

As discussed previously, both manakins and zebra finches express AR in the spinal cord and several skeletal muscles, with expression in manakins greatly exceeding that of zebra finches and a host of other species that do not perform extravagant physical displays [41, 44]. Other work suggests that activation of these AR populations regulates motor skills that are necessary for male manakins to perform their complex display maneuvers [46]. In this framework, our current results suggest that transcription of the enzymes that metabolize androgenic hormones are modified in the manakin neuro-motor system to optimize androgenic signaling. For example, we find that male golden-collared manakins express greater levels of type 1 5α-reductase, relative to females. At the same time, golden-collared manakins also express lower levels of 5β-reductase compared to zebra finches. Taken together, these data suggest that male manakins have a greater capacity to locally enhance androgen action in the spinal cord and wing muscles not only by increasing metabolic production of active 5α-DHT, but also by suppressing metabolic inactivation of androgen into 5β-DHT.

Notably, we find a variety of muscle-specific effects in enzyme transcription. For example, in zebra finch muscles, type 1 5α-reductase 1 was expressed at significantly higher levels in the male SH compared to females, with no sex difference in other muscles. These results indicate that males exceed females in the capacity to provide 5α-reduced androgenic metabolites to AR in some passerine skeletal muscles, a result predicted by our initial hypothesis. Furthermore, in contrast to our hypothesis, male zebra finches expressed more 5β-reductase in the SH than females, but there was no sex differences in the expression of this gene in the other muscles. This level of muscle specificity in enzyme expression was not present in golden-collared manakins; thus, even though both type 1 5α-reductase and 5β-reductase can be expressed in some wing muscles at greater levels in males than in females, it is clear that such effects are not consistent across species. The functional significance of these muscle-specific sex differences in androgenic reducing ability within the zebra finch muscular system is currently unclear.

Across all regions of the manakin spinal cord, type 1 5α-reductase was expressed at greater levels in males compared to females. By contrast, no differences across sex were observed in zebra finches type 1 5α-reductase, and there were no sex differences for type 2 in either manakins or zebra finches. Moreover, there were no sex differences in 5β-reductase expression in the spinal cord of either species, although golden-collared manakins expressed far less of this enzyme than zebra finches. These results suggest that the type 1 5α-reductase isoform may be especially important in the male manakin spinal cord and that relatively low levels of 5α-reductase may exacerbate the capacity to metabolize androgens into 5α-DHT (see above). Interestingly, we have previously observed that after injection of radiolabeled T, radioactivity accumulates to a greater degree in the spinal cords of male manakins, compared to females [48]. Thus, the combination of the male-specific profile of elevated type 1 5α-reductase and diminished 5β-reductase in the species may account for the clear sex differences in androgenic occupation of spinal AR. To this end, because androgens clearly impact the performance of the complex masculine courtship behaviors of golden-collared manakins by acting on both motor and sensory neurons in the spinal cord [47], elevated expression of type 1 5α-reductase likely plays a crucial role in this behavioral activation.

### General considerations and limitations

Factors other than courtship behavior may account for some of the differences in reductase expression we uncover. For example, in theory, species and sex differences in circulating androgens may influence the expression of reductase enzyme [56]. We can rule out this possibility, given that males used in this study were actively courting females and maintained enlarged testes at the time of dissection. This indicates that circulating androgens were elevated in these individuals enough to activate reproduction [51, 52]. Moreover, testosterone levels were likely basal in females, given that circulating androgens are often undetectable in female manakins and zebra finches during the breeding season [51, 53]. If circulating androgens do in fact impact reductase mRNA levels in the muscle and spinal cord, then these effects are certainly not robust. Zebra finch muscles and spinal cord tissue are far less sensitivity to androgens than manakins [41, 44], but zebra finches tend to express greater amounts of 5α- and 5β-reductase mRNA as manakins. Had circulating testosterone driven the majority of the variation that we detected, we would have expected to see the opposite relationship. Similarly, we detected relatively few sex differences in zebra finch reductase expression, which we would otherwise expect if androgens were the main driver of reductase production.

Equally important to recognize are some of the limitations of our species comparison. Golden-collared manakins and zebra finches are distantly related within the passerine family, and thus a variety of ecological, social, and neutral evolutionary processes may influence the evolution of peripheral androgenic control systems [57]. Still, we know that strong sexual selection operates on the wing neuro-motor apparatus to support the golden-collared manakin’s acrobatic display movements [58], and that androgenic signaling mechanisms are a primary target of this selection [44]. Thus, it is reasonable to assume that the species differences we uncover in androgenic metabolic capability are in large part due to differences in courtship behavior.

### A broader functional role of reductase enzymes

In general, expression levels of the two isoforms of 5α-reductase were similar enough to each other to suggest that neither isoform exerted the predominant or exclusive functional effect. Thus, while patterns of expression indicate that the type 1 isoform may be locally regulated to enhance formation of more potent 5α-reduced metabolites in manakin skeletal muscle and spinal cord, the type 2 isoform would appear abundant enough in both species to similar have some effect on androgenic metabolism. These differences may stem from the possibility that the two isoforms utilize different substrates with different functional endpoints across species, and this idea merits further investigation in birds.

Androgen-reducing enzyme may also have other physiological functions in manakins and zebra finches. For example, in addition to androgens, some progestins can be 5α-reduced producing physiologically relevant products. One of the best examples of this is allopregnanolone, which is an endogenous steroid that potently facilitates GABAergic inhibition and is produced via 5α-reduction and 3α–hydroxylation of progesterone [59]. It is therefore possible that in the spinal cord, where GABA_A_ receptors are expressed, locally produced allopregnanolone might regulate neurotransmission [60]. Elevated levels of type 2 5α-reductase in zebra finches might therefore reflect greater action of allopregnanolone in this species. Interestingly, allopregnanolone can also exist as the 5β-reduced isoform; thus, because 5β-reductase is also expressed at greater levels in the zebra finch spinal cord, the 5β-reduced form of this hormone may potentiate GABAergic transmission in zebra finches with equal, if not even greater, efficacy than the 5α-reduced isoform [61]. We have no evidence for 3α-hydroxysteroid dehydrogenase in avian spinal cord it has been detected in the spinal cord of rodents [60]. Thus, it remains possible that these reactions occur to a greater degree in zebra finches than in manakins to regulate inhibitory neurotransmission. Perhaps the balance between inhibitory and excitatory neurotransmission in the spinal cord favors steroid-dependent inhibitory mechanisms is species with a lower degree of androgen-dependent motor function.

There is also recent evidence that 5α-reductase can metabolize glucocorticoids, thus limiting their actions [62]. Therefore, we cannot dismiss the possibility that these enzymes may participate in ameliorating stress hormone actions on these neuromuscular tissues in both avian species.

## Conclusions

In summary, our results demonstrate that type 1 and type 2 5α-reductase, as well as 5β-reductase, are expressed in skeletal muscle and spinal cords of two passeriform species, the sub-oscine golden-collared manakin and the oscine zebra finch. Patterns of expression in these tissues and between these species are consistent with a role for metabolic regulation of androgenic hormones within the neuro-motor apparatus to influence courtship behavior, especially the golden-collared manakin’s physically demanding courtship display. Whereas there is debate as to whether 5α-reductase plays a significant role the activation of AR in mammalian skeletal muscle [29–32], our results suggest that the case may be different in birds. Future work that evaluates androgen-binding and AR-dependent gene expression in the presence of absence of 5α-reductase inhibitors in this species, and perhaps others, are now warranted.

## Abbreviations

AR, androgen receptor; PEC, pectoralis; SC, surprcoricoideus; SH, scapulohumeralis caudalis; T, testosterone
